# Induction of Regulatory T Cells by High-Dose gp96 Suppresses Murine Liver Immune Hyperactivation

**DOI:** 10.1371/journal.pone.0068997

**Published:** 2013-07-18

**Authors:** Xinghui Li, Zhen Liu, Xiaoli Yan, Xiaojun Zhang, Yang Li, Bao Zhao, Shengdian Wang, Xuyu Zhou, George F. Gao, Songdong Meng

**Affiliations:** 1 Key Laboratory of Pathogenic Microbiology and Immunology, Institute of Microbiology, Chinese Academy of Sciences, Beijing, China; 2 University of Chinese Academy of Sciences, Beijing, China; 3 Key Laboratory of Infection and Immunity, Institute of Biophysics, Chinese Academy of Sciences, Beijing, China; Wayne State University, United States of America

## Abstract

Immunization with high-dose heat shock protein gp96, an endoplasmic reticulum counterpart of the Hsp90 family, significantly enhances regulatory T cell (Treg) frequency and suppressive function. Here, we examined the potential role and mechanism of gp96 in regulating immune-mediated hepatic injury in mice. High-dose gp96 immunization elicited rapid and long-lasting protection of mice against concanavalin A (Con A)-and anti-CD137-induced liver injury, as evidenced by decreased alanine aminotransaminase (ALT) levels, hepatic necrosis, serum pro-inflammatory cytokines (IFN-γ, TNF-α, and IL-6), and number of IFN-γ ^+^ CD4^+^ and IFN-γ ^+^ CD8^+^ T cells in the spleen and liver. In contrast, CD4^+^CD25^+^Foxp3^+^ Treg frequency and suppressive function were both increased, and the protective effect of gp96 could be generated by adoptive transfer of Treg cells from gp96-immunized mice. *In vitro* co-culture experiments demonstrated that gp96 stimulation enhanced Treg proliferation and suppressive function, and up-regulation of Foxp3, IL-10, and TGF-β1 induced by gp96 was dependent on TLR2- and TLR4-mediated NF-κB activation. Our work shows that activation of Tregs by high-dose gp96 immunization protects against Con A- and anti-CD137-induced T cell-hepatitis and provides therapeutic potential for the development of a gp96-based anti-immune hyperactivation vaccine against immune-mediated liver destruction.

## Introduction

Regulatory T cells (Tregs) are a subset of CD4^+^ T cells that highly express IL-2Rα chain (CD25), and their differentiation and function are controlled by the forkhead/winged helix transcription factor (Foxp3) [1]. The activation and suppressive function of Tregs require TCR signaling and stimulation by TGF-β and IL-2 [2]. Tregs suppress the activation, proliferation, diﬀerentiation, and eﬀector functions of many cell types, including CD4^+^ and CD8^+^ T cells, and play a major role in maintaining immune homeostasis and immune tolerance to self-Ag, as well as to pathogens and tumors [3–5]. Suppression of conventional T cells by Tregs involves immunosuppressive soluble factors (e.g., TGF-β and IL-10) and cell-cell contact [6]. Due to their potent immune regulatory phenotypes, manipulation of Treg cell activity provides huge therapeutic potential to restrain immune hyperactivation in autoimmunity, inflammation, and allograft rejection [2,7,8].

The immunomodulatory role of Tregs in hepatitis B virus (HBV) infection with different disease stages has been extensively studied. Higher frequencies of Tregs are observed in chronic hepatitis B (CHB). They suppress viral-specific T cell responses and play a key role in maintenance of immune tolerance to HBV and viral persistence [4,9]. On the other hand, as hepatic T lymphocytes- and NK cells-mediated inflammation is involved in the pathogenesis of HBV-induced chronic liver diseases [10–12], Tregs may play a key role in intrahepatic immune regulation. Treg frequency has been shown to be inversely correlated with immune-mediated liver injury and pathogenesis of HBV-associated fibrosis progression and liver failure [13,14], and the decline of HBcAg peptide-specific Treg cells may partially account for acute exacerbation of CHB [15,16], though the exact mechanisms of Treg function await further investigation. Furthermore, a study performed in a mouse model of acute HBV infection demonstrates that Tregs restrain immune-mediated liver damage by suppressing effector T cells via inhibition of cytokine production and cytotoxicity at the cost of delaying virus clearance [17].

Concanavalin A (Con A)-induced liver damage in the mouse is a well-characterized model of T cell-dependent experimental liver injury [18], and has been used to investigate the immune-mediated pathology of autoimmune hepatitis and viral hepatitis. Intravenous injection of mice with Con A activates T lymphocytes and induces secretion of Th1 cytokines, and leads to symptoms of acute hepatitis, including lymphocyte infiltration in the liver, hepatocyte necrosis and elevated ALT levels, which resemble the pathophysiology of T cell-mediated hepatic injury and liver diseases [19–21].

Heat shock protein gp96, the endoplasmic reticulum form of Hsp90, plays an important role in modulating innate and adaptive immunity [22,23]. Owing to its unique immunogenicity, clinical trials have been initiated using autologous gp96-peptide complexes for treatment of cancers [24]. In our previous study, we used titrated doses of gp96 (0, 0.5, 5, 10, 20, 50, 100 and 200 µg/mice) for immunization and found that 10-20 µg of gp96 elicited the highest CTL responses, but a dramatic decrease in CTL activity was observed when the immunization dose increased to 50-100 [25]. We and others have further demonstrated that activation of CTL at low dose of gp96 is far more pronounced than Treg activation, however, high-dose gp96 immunization of mice significantly enhances Treg frequency and suppressive function, which eventually abrogates gp96-induced T cell activation, and that gp96 potentiates Treg suppressive function (at least partially) via TLR4 [25,26]. Given the key roles of Tregs in maintaining immune tolerance to prevent inflammation-induced pathogenesis, we aimed to investigate the potential role and mechanism of gp96-induced Tregs in regulating immune-mediated hepatic injury in this study.

## Materials and Methods

### Ethics Statement

All animal procedures were conducted in accordance with “the regulation of the Institute of Microbiology, Chinese Academy of Sciences of Research Ethics Committee,” The protocol was approved by the Research Ethics Committee of the Institute of Microbiology, Chinese Academy of Sciences (Permit Number: PZIMCAS2012004).

### Mice

Female BALB/c mice (6- to 8-weeks-old) were purchased from the Peking University laboratory animal center (Beijing, China). Foxp3-EGFP transgenic mice were purchased from the Jackson Laboratory. Mice deficient for Myd88 gene (Myd88-/-) were maintained in Beijing Laboratory Animal Research Center.

### Preparation of mice gp96, GST-gp96, and FITC-gp96

Purification of gp96 protein from mouse liver and removal of endotoxin was performed as previously described [25,27]. The possible contamination of Con A in gp96 preparations during gp96 purification procedure with Con A-Sepharose affinity chromatography was ruled out by detection of Con A levels using Plant Con A Elisa kit (DongSongs Bio, Beijing, China), and no residual Con A was detected in the purified gp96 protein. The concentration of endotoxin, measured using the Limulus Amebocyte Lysate assay (BioWhittaker, Walkersville, MD), in gp96 preparations was ultimately reduced to 1 EU/mg protein. GST-fused gp96 was expressed in *Escherichia coli* and purified by passing the lysates through a glutathione Sepharose 4 fast flow column. FITC-gp96 was labeled with a FITC Protein Labeling Kit (Invitrogen, Carlsbad, CA) according to the manufacturer’s instructions.

### Immunization of mice and induction of hepatitis

Female BALB/c mice were immunized s.c. with 100 µg gp96 or 100 µg mouse serum albumin (MSA) as control three times at 0, 1, and 3 weeks. Mice were given a single i.v. injection of Con A at 15 µg/g of body weight 7 d after the last immunization. Serum alanine aminotransferase (ALT) levels were determined at the indicated time. Or, BALB/c mice immunized with gp96 for three times received a Con A injection at 1, 2, or 3 months respectively after the last immunization to determine the longevity of gp96-induced protective effect. Or, BALB/c mice immunized with 100 µg gp96 or MSA for one time received a Con A injection at 4, 7, 10, or 13 days respectively after immunization to determine how fast gp96 immunization could protect mice from Con A-induced liver injury. ALT levels were determined 12 h post-injection. Or mice immunized with gp96 or MSA for three times were injected i.p. with 100 µg anti-CD137 mAb (clone 2A, rat IgG2a) 7 d after the last immunization. ALT levels were measured 10 d post-immunization. At the start of immunization the mice were 6-8 weeks old. Mice immunized with 100 µg MSA served as control group. Each group contained at least 5 mice. Serum ALT levels were determined with a commercial kit (Roche Diagnostics, Mannheim, Germany) using 10 µl serum and are expressed as U/L.

### Histological examination

Liver tissues from mice were fixed with 10% phosphate-buffered formalin and embedded in paraffin. Consecutive sections of 5µm thickness were deparaffinized and stained with H&E. The necrosis areas (loss of architecture and vacuolization) were quantified by measuring the areas of necrosis in six separate fields using NIH ImageJ 1.46 software. The percentage of liver necrosis was calculated as (total areas of necrosis)/(total areas of the six fields) × 100%.

### Isolation of splenocytes and liver inﬁltrating lymphocytes (LILs)

Mice were sacrificed 1 week after the last immunization or 12 h after the Con A injection. Splenocytes were isolated as previously described [25]. The LILs were prepared as described [28].

### Cell purification and adoptive cell transfer

CD4^+^CD25^+^ T cells were purified from splenocytes using a CD4^+^CD25^+^ regulatory T cell isolation kit (Miltenyi Biotec, Auburn, CA) according to the manufacturer’s instructions. CD4^+^ T cells were pre-enriched by depletion of non-CD4^+^ T cells using the regulatory T cell isolation kit. Foxp3-EGFP^+^ Tregs were sorted by CD4, CD25, and EGFP marker using a BD FACSAria machine. CD4^+^CD25^+^ Tregs (1×10^6^), Foxp3-EGFP^+^ Tregs (1×10^6^), CD4^+^ T cells (1×10^7^), or CD4^+^CD25^-^ T cells (1×10^7^) were injected i.v. into mice, followed by i.v. injection of Con A.

### ELISA assay

Cytokines (IFN-γ, TNF-α, IL-6, and IL-10) in serum or splenocyte culture supernatants were measured using ELISA kits (eBioscience, San Diego, CA) according to the manufacturer’s instructions.

### Co-immunoprecipitation

Equal amounts of TLR Abs or control Ab were added to Treg lysates and incubated overnight at 4^o^C. The supernatant was incubated with protein G beads for another 2 h, followed by extensive washing of the beads. Protein G beads were boiled in SDS-PAGE sample buffer to elute the immunoprecipitates for detection of gp96 by Western blotting.

### GST pull down assay

GST-gp96 or GST was mixed with Treg lysates and incubated overnight at 4^o^C. Glutathione Sepharose medium was added and incubated for 2 h. The beads were washed three to five times and boiled in SDS-PAGE sample buffer for immunoblotting of TLR2 and TLR4.

### *In vitro* suppression assay

CD4^+^CD25^-^ effector T cells (Teff) were labeled with a CFSE Cell Proliferation Kit (Invitrogen, Carlsbad, CA) according to the manufacturer’s instructions. In 96-well round-bottom plates, 3×10^5^ Teff were co-cultured with gp96-pre-treated or untreated Tregs at a ratio of 3:1 in the presence of 1 µg/ml anti-CD3 and 1 µg/ml anti-CD28 at 37^o^C for 72 h. The suppression rate was calculated as [CFSE^low^ (Teff without Treg)-CFSE^low^ (Teff with Treg)]/CFSE^low^ (Teff without Treg) ×100%.

### Quantitative real-time PCR

Splenocytes were stimulated *in vitro* with 100 µg/ml gp96 in the presence or absence of 30 µg/ml TLR2 and TLR4 inhibitor OxPAPC (InvivoGen, San Diego, CA) or 1 µM NF-κB inhibitor BAY 11-7082 (Sigma, St. Louis, MO) (pre-treated for 20 min) for 24 h. Total RNA was extracted using Tripure Isolation Reagent (Roche Diagnostics, Mannheim, Germany) according to the manufacturer’s recommendations. The mRNA levels of Foxp3, IL-10, TGF-β1, and β-Actin were analyzed by quantitative real-time PCR using SYBR Premix Ex Taq (Takara, Dalian, China). Target gene expression was normalized to β-Actin mRNA levels. All samples were run in triplicate.

### Cytochemical staining of NF-κB

Raw264.7 cells were grown on slides. Cells were stimulated with 100 µg/ml gp96 for 30 min, washed with PBS, permeabilized, blocked, and incubated with 5 µg/ml anti–NF-κB/p65 (Santa Cruz). Specific secondary anti-IgG antibodies labeled with FITC were applied. Cell nuclei were stained with DAPI (Invitrogen).

### Statistics

The results are shown as means ± SEM. Statistical analysis was performed using GraphPad Prism 5.01. A *p* value < 0.05 was considered statistically significant.

## Results

### High-dose gp96 immunization protects mice from Con A- and anti-CD137 agonist mAb-induced liver injury

**Figure 1:**
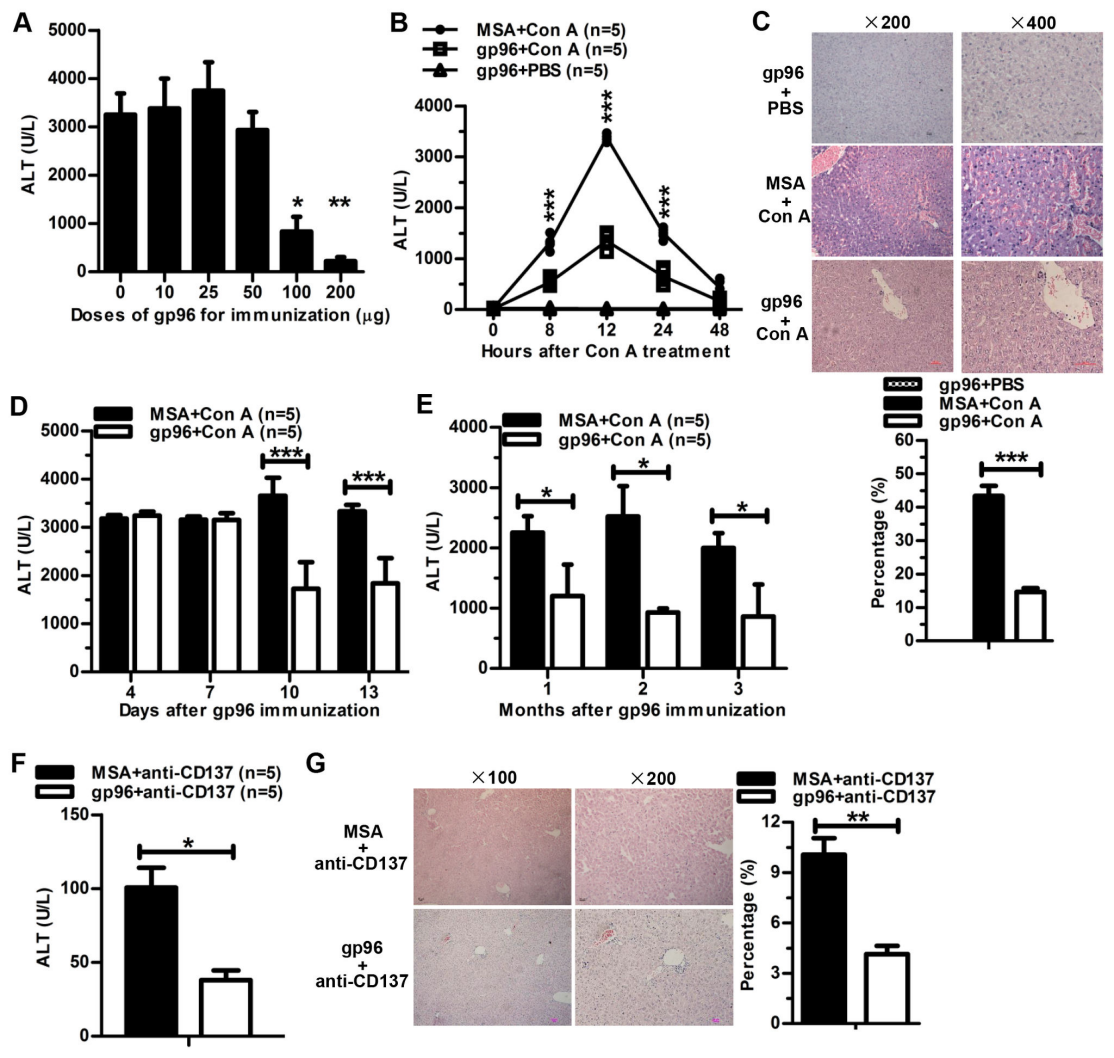
High-dose gp96 immunization protects mice from Con A- and anti-CD137 mAb-induced liver injury. (A) BALB/c mice were immunized with the indicated doses of gp96. Serum ALT levels were measured 12 h after Con A injection (* and ** compared to mice immunized with 0, 10, 25 or 50 µg gp96). Detection of serum ALT levels (B) and histological examination of the liver (C) of gp96-immunized or MSA-immunized (as control) mice injected with or without Con A. Percentages of hepatic necrosis in liver sections were calculated and are shown (C). (D) Mice with a single immunization of gp96 were injected with Con A at the indicated time points. Serum ALT was measured 12 h after injection. (E) ALT levels of gp96-immunized mice injected with Con A at 1, 2, or 3 months after gp96 immunization. Detection of serum ALT levels (F) and histological examination of the liver (G) of gp96-immunized or MSA-immunized mice 10 d after injection of anti-CD137 mAb. *, *p* < 0.05; **, *p* < 0.01; ***, *p* < 0.001. Data are representative of two independent experiments.

We first examined whether high-dose gp96 immunization was able to protect mice from Con A-mediated liver injury. BALB/c mice were immunized s.c. with different doses of gp96 at 0, 1, and 3 weeks, and at 7 d after the last immunization, mice received a single i.v. injection of Con A. High doses (100-200 µg/mice) of gp96 could significantly reduce liver injury as determined by serum ALT levels at 12 h after Con A treatment, whereas low doses (10-50 µg/mice) of gp96 could not (Figure 1A). Based on these preliminary data, we chose 100 µg of gp96/mice as a high dose for further investigation. High dose of gp96 treatment significantly reduced liver injury at different time points after Con A treatment (Figure 1B). Twelve hours after Con A injection, serum ALT levels peaked in both gp96-treated and untreated mice, and compared to control mice, gp96-treated mice displayed approximately 3-fold lower ALT activity. Similar results were observed by liver histological analysis (Figure 1C). Liver tissue of gp96-immunized mice displayed dramatically less areas (66%) of necrosis with the signature of loss of architecture and vacuolization than control mice. To determine how fast gp96 immunization elicits such protection, mice received a single dose of gp96 immunization were given a Con A injection at different time points. Protection appeared as early as 10 d after gp96 administration (Figure 1D). To determine the longevity of the protective effect, mice immunized with gp96 three times received a Con A injection at 1, 2, or 3 months after the last immunization, respectively. As shown in Figure 1E, gp96 treatment significantly reduced Con A-liver injury for at least 3 months.

To further test the capability of gp96 to attenuate immune-mediated liver pathology, a mouse model of anti-CD137 agonist mAb-induced liver inflammation was employed. CD137 (4-1BB or TNFR9) is a surface glycoprotein expressed on activated T cells, NK cells, dendritic cells, and macrophages, and the CD137 agonistic mAb induces CD8^+^ T cell-mediated liver inflammation in mice [29]. BALB/c mice were injected i.p. with 100 µg anti-CD137 mAb 7 d after the third immunization with gp96. Pronounced protective effects were observed by gp96 immunization as determined by serum ALT levels (Figure 1F) and liver histological analysis (Figure 1G) 10 d after anti-CD137 injection.

### Gp96 immunization suppresses Con A-induced immune hyperactivation by induction of CD4^+^CD25^+^Foxp3^+^ Tregs

**Figure 2:**
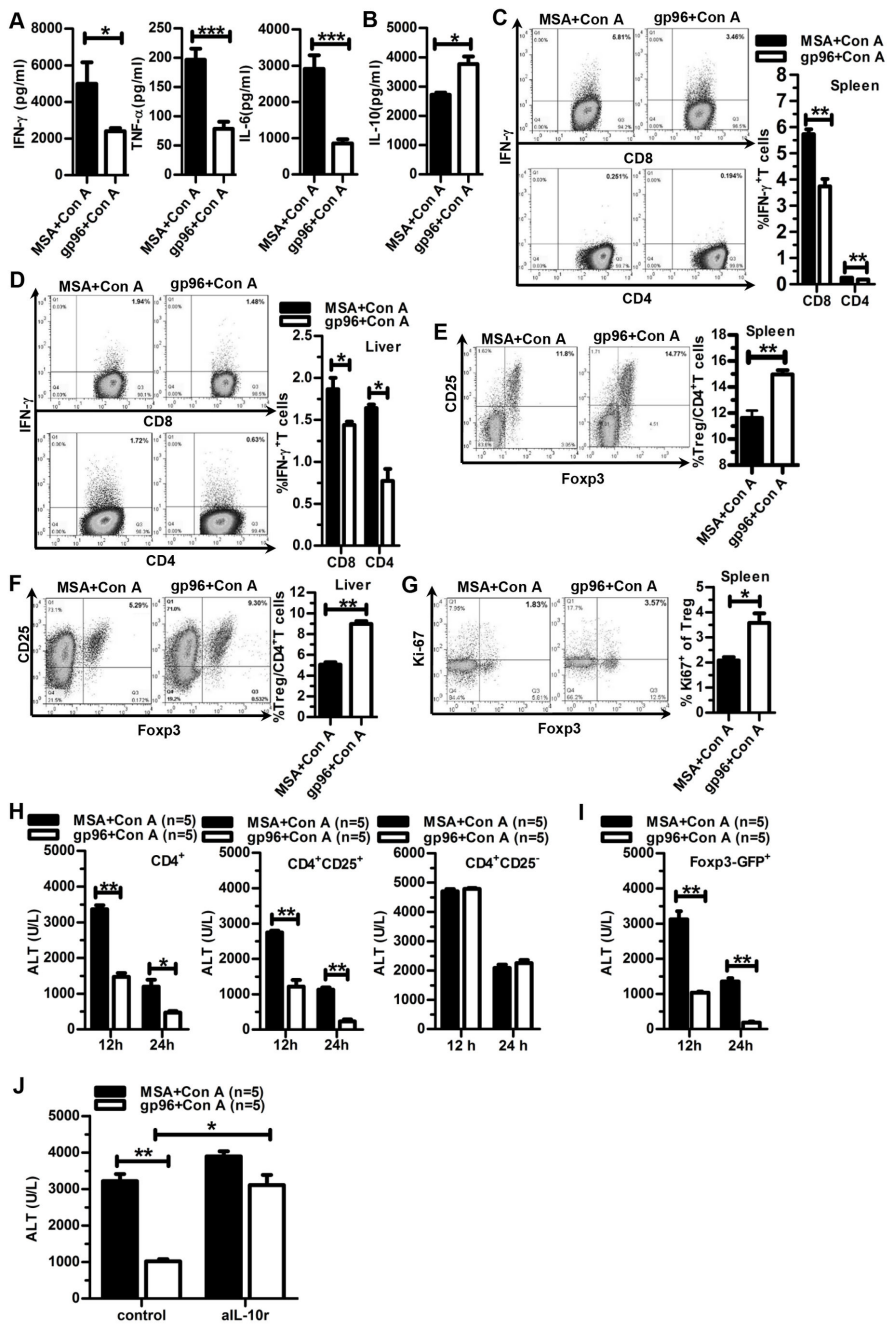
Gp96 immunization inhibits Con A-induced immune hyperactivation by induction of Tregs. BALB/c mice immunized with gp96 three times were challenged with Con A. At 12 h after the Con A challenge, serum levels of pro-inflammatory cytokines (A) and IL-10 (B) were measured by ELISA. Flow cytometric analysis was performed to determine the frequency of IFN-γ ^+^ CD8^+^ and IFN-γ ^+^ CD4^+^ T cells in the spleen (C) or liver (D), CD4^+^CD25^+^Foxp3^+^ Tregs in the spleen (E) or liver (F), and expression of Ki-67 by Tregs in the spleen of mice (G). (H) Different subtypes of T cells from gp96-immunized mice (the bulk of total CD4^+^ population, and the sorted CD4^+^CD25^+^ and CD4^+^CD25^-^ subpopulations) were adoptively transferred to naïve mice as indicated, and 24 h later, the mice were challenged with Con A. At 12 h after Con A treatment, serum ALT levels were measured. (I) Foxp3-EGFP^+^ Tregs were sorted by FACS from gp96-immunized Foxp3-EGFP transgenic mice and were adoptively transferred to naïve recipient mice. Serum ALT levels were measured 12 h after Con A injection. (J) Mice immunized with gp96 or MSA were i.p. injected with 250 µg of mice anti-IL-10 receptor antibody (aIL-10r) or control antibody one day prior to Con A injection. Serum ALT levels were detected 12 h after Con A treatment. *, *p* < 0.05; **, *p* < 0.01; and ***, *p* < 0.001. Data are representative of two independent experiments and FACS analyses were performed for at least three times in each experiment.

Mice immunized with 100 µg gp96 were challenged with Con A, and key serum pro-inflammatory cytokines [19] were measured by ELISA 12 h after challenge. Compared to the control (MSA), gp96 immunization decreased IFN-γ, TNF-α, and IL-6 levels by 52, 60, and 71%, respectively (Figure 2A). In contrast to the pro-inflammatory cytokines tested, the anti-inflammatory cytokine IL-10 significantly increased in gp96-immunized mice (Figure 2B). Because Con A-induced liver injury is associated with T cell-dependent inflammation [18–21], we next investigated the T cell profile of the spleen and liver. Both the number of IFN-γ ^+^ CD4^+^ and IFN-γ ^+^ CD8^+^ T cells in the spleen (Figure 2C) and liver (Figure 2D) were significantly lower in gp96 immunized mice relative to controls. As expected, the CD4^+^CD25^+^Foxp3^+^ Treg frequency in the spleen (Figure 2E) and liver (Figure 2F) significantly increased by 29 and 78%, respectively, after gp96 immunization. In addition, a larger proportion of Tregs were active in the cell cycle in gp96-immunized mice compared to control mice, as measured by Ki-67 staining (Figure 2G). However, no differences in the total LIL number and T cell frequency of LILs were observed between gp96-immunized and control mice (data not shown). Importantly, the increase of serum ALT levels by Con A treatment was significantly suppressed in recipients of either adoptively transferred CD4^+^ T cells or CD4^+^CD25^+^ Tregs but not CD4^+^CD25^-^ T cells from gp96-immunized mice (the number of CD4^+^ and CD4^+^CD25^-^ cells was ten times more than that of CD4^+^CD25^+^ cells), as compared to control mice (Figure 2H). As Foxp3 is a more specific marker for Tregs, we further confirmed the protective effect of gp96-activated Tregs by adoptively transferring FACS-sorted CD25^+^Foxp3-EGFP^+^ cells from gp96- or MSA-immunized Foxp3-EGFP transgenic mice (Figure 2I). Moreover, as shown in Figure 2J, IL-10 blockade largely abrogated the protection of gp96 immunization against Con A-induced liver injury. These data indicate that inhibition of immune hyperactivation is due to increased Treg activity induced by gp96.

### High-dose gp96 enhances Treg proliferation and function by up-regulation of Foxp3 expression and IL-10 secretion

**Figure 3:**
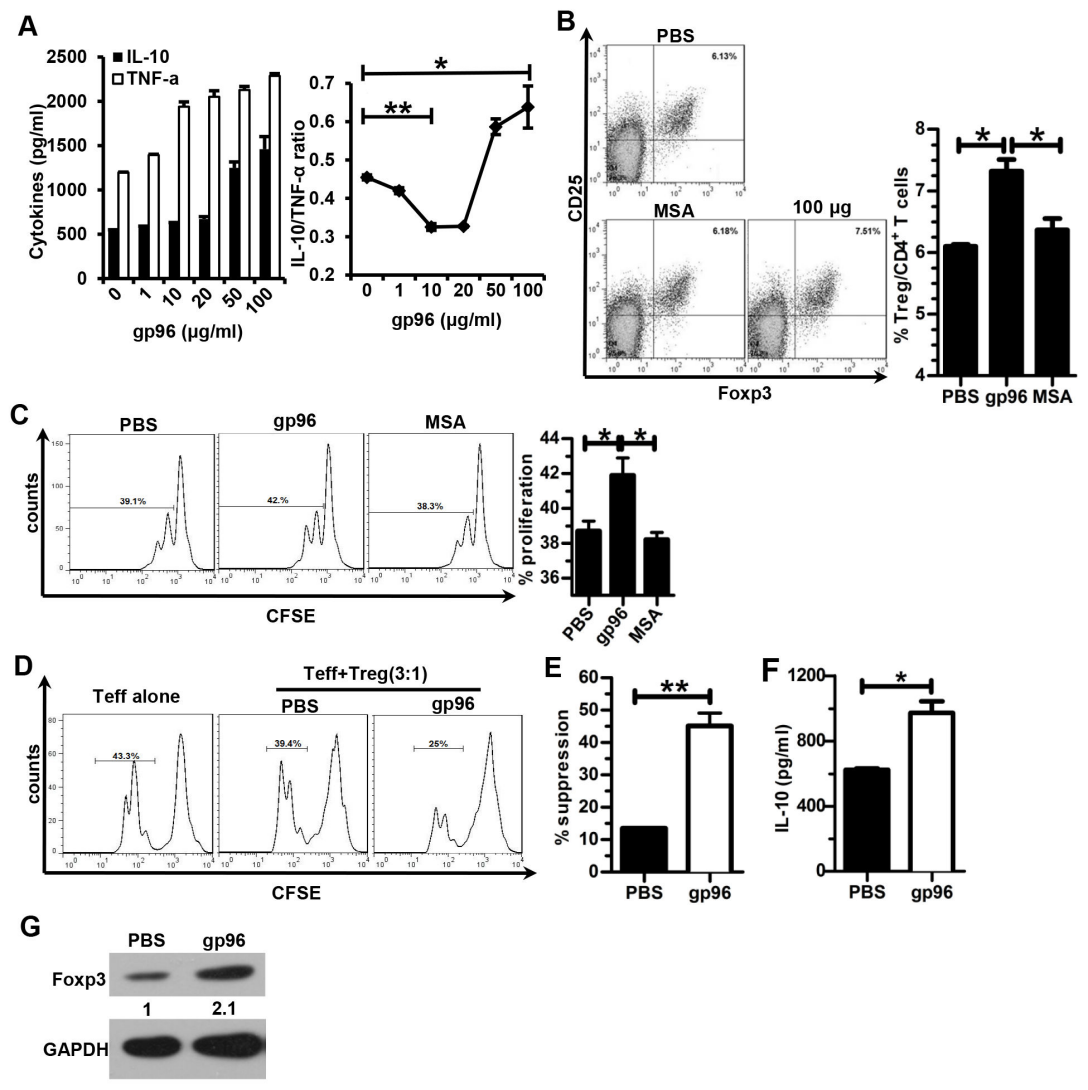
High-dose gp96 promotes Treg proliferation and function by up-regulation of Foxp3 expression and IL-10 secretion. (A) Mice splenocytes cultured in the presence of 1 µg/ml anti-CD3, anti-CD28 Abs and 50 IU/ml IL-2 were stimulated with increasing amounts of gp96 for 24 h, and the TNF-α and IL-10 levels in the supernatants were analyzed by ELISA. Mice splenocytes (B–E) or Treg cells (F, G) were incubated with 100 µg/ml gp96 *in vitro* in the presence of anti-CD3 and anti-CD28 Abs and IL-2. The Treg frequency was measured by FACS after 48 h (B). Proliferation of Tregs was determined by CSFE staining 3 d later (C). A total of 3×10^5^ CSFE-labeled CD4^+^CD25^-^ Teff cells were cultured with Tregs at a ratio of 3:1 for 3 d. The Teff cell division cycle was measured by FACS (D). The suppression rate for proliferation was calculated (E). The IL-10 levels in the supernatants of Tregs were measured by ELISA (F). The Foxp3 expression of Tregs was detected by Western blotting (G). *, *p* < 0.05; **, *p* < 0.01. Results are representative of three independent experiments and FACS analyses were performed for at least five times in each experiment.

Because the balance between inflammatory and anti-inflammatory cytokines controls the direction of the immune response [30,31], we first examined the TNF-α and IL-10 expression levels by ELISA in the supernatants of splenocytes stimulated with increasing amounts of gp96 for 24 h. As shown in Figure 3A, gp96 at low concentrations (10-20 µg/ml) stimulated TNF-α expression, whereas at high concentrations (50-100 µg/ml), it preferably increased IL-10 levels. The ratio of IL-10 to TNF-α dropped under treatment with low dose of gp96 (*p* < 0.01), and peaked under high-dose gp96 treatment (*p* < 0.05).

As shown in Figure 3B, the number of Treg cells increased with high-dose gp96 treatment. Gp96 significantly promoted the proliferation of Foxp3^+^ Tregs, as compared to PBS or MSA treatment (Figure 3C). Moreover, Tregs pre-incubated with 100 µg/ml gp96 significantly suppressed the proliferation of CD4^+^CD25^-^ effector T cells (Teff), but a much lower extent of suppression was observed using non-pre-incubated Tregs (Figure 3D). The suppressive rate of gp96-stimulated Tregs was much higher than that of unstimulated Tregs (*p* <0.01) (Figure 3E). Further, there was a significant increase of IL-10 secretion in isolated Tregs stimulated with high-dose gp96 (Figure 3F). Notably, treatment of Treg cells with gp96 resulted in an approximately 2-fold increase in Foxp3 expression compared to untreated cells (Figure 3G).

### Gp96 interacts with TLRs to activate NF-κB signaling in Tregs

**Figure 4:**
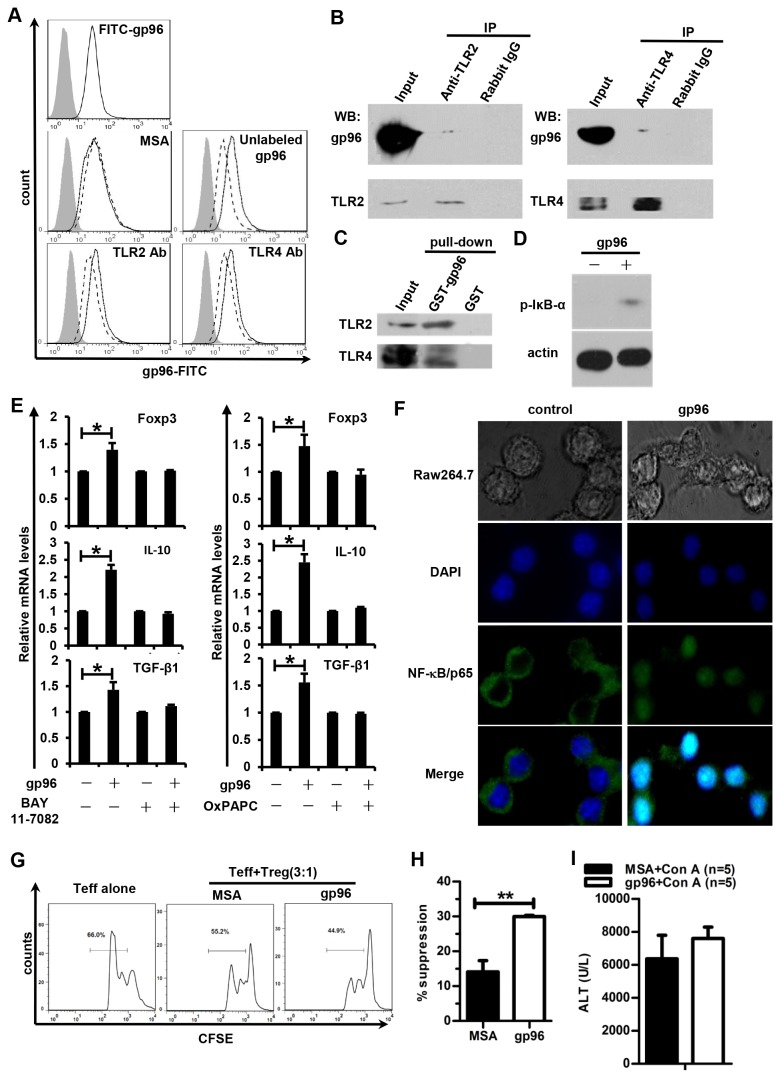
Gp96 activates NF-κB signaling through interaction with TLR2 and TLR4 on Tregs. (A) The binding of FITC-gp96 (solid line) to Tregs in the presence of unlabelled gp96 or TLR2 or TLR4 Abs (dashed line). Tregs only are indicated by the gray histogram. (B) Co-immunoprecipitation of gp96 and TLR2 or TLR4 in Tregs. (C) GST-gp96 could pull down TLR2 and TLR4 in Treg cell lysates. (D, E) Treg cells were incubated with 100 µg/ml gp96 *in vitro*. The phospho-IκB-α levels were detected by Western blotting 30 min later (D). The mRNA levels of Foxp3, IL-10, and TGF-β1 with or without OxPAPC or BAY 11-7082 pre-treatment were analyzed by real-time PCR after 24 h (E). (F) Raw264.7 macrophages were stimulated with 100 µg/ml gp96 for 30 min, and NF-κB was detected by immunofluorescent staining. Magnification ×400. (G) BALB/c mice were immunized one time with 100 µg of gp96 and Tregs were isolated 24 h after immunization to perform *ex vivo* suppressive function analysis as described in Figure 3D. The suppressive rate of proliferation was calculated (H). (I) Myd88-/- mice immunized with gp96 three times were challenged with Con A. At 12 h after the Con A challenge, serum ALT levels were measured. *, *p* < 0.05. Results are representative of three independent experiments and FACS analyses were performed for at least five times in each experiment.

Because TLR2 and TLR4 ligands and agonists modulate Treg proliferation, survival, and function by directly acting on the Treg population [26,32] and gp96 interacts with TLRs and activates innate immunity [33], we decided to investigate the modulation of Tregs by gp96 via TLR2 and TLR4 on the surface of Tregs. Freshly isolated Tregs were incubated with FITC-labeled gp96 with or without excess unlabeled gp96 or an irrelevant protein. FACS analysis revealed that Tregs are able to bind FITC-gp96 (Figure 4A, upper panel), and the binding was specific because it could be competed by unlabeled gp96 but not MSA (Figure 4A, middle panels). Importantly, the presence of TLR2 Ab or TLR4 Ab interfered with FITC-gp96 binding on Tregs (Figure 4A, lower panels), indicating that gp96 binds to TLR2 and TLR4 on Tregs. Moreover, gp96 could be co-precipitated with TLR2 and TLR4 (Figure 4B), which was reconfirmed by GST-gp96 pull-downs of TLR2 and TLR4 (Figure 4C). The NF-κB signaling pathway was also activated in isolated Treg cells stimulated with gp96, as indicated by Western blotting of phospho-IκB-α (Figure 4D).

We measured the Foxp3, IL-10, and TGF-β1 gene transcription levels of Tregs after gp96 stimulation. The levels of all three mRNAs were up-regulated with gp96 stimulation (Figure 4E). Pre-treatment of Tregs with Bay 11-7082, a specific inhibitor of the IκB kinase IKK, or OxPAPC, a TLR signaling inhibitor that is restricted to TLR2 and TLR4 [34], almost totally abrogated the up-regulation of Foxp3, IL-10, and TGF-β1 mRNA levels induced by gp96. Activation of NF-κB signaling by gp96 was confirmed in gp96-stimulated Raw264.7 macrophage cells with nuclear translocation of NF-κB (Figure 4F). As TLR-mediated activation could be very rapid, we then examined the *ex vivo* function of Tregs 24 h after gp96 immunization. The proliferation of Teff incubated with Tregs from gp96-immunized mice was much lower than control mice (Figure 4G). High-dose gp96 immunization significantly increased Treg suppression of Teff proliferation as quick as 24 h post-immunization (Figure 4H). In order to further confirm that TLR2 and TLR4 are involved in gp96-induced Treg activation *in vivo*, Myd88-/- mice were immunized with gp96 to determine the protection effect against Con A-induced liver injury. As shown in Figure 4I, the protective effect of high-dose gp96 immunization was completely abolished in TLR2 and TLR4 signal deficient mice. Together, the results demonstrate that elevated transcription levels of Foxp3, IL-10, and TGF-β1 by gp96 occurred via a TLR2- and TLR4-dependent NF-κB-mediated signaling pathway.

## Discussion

Although there is emerging evidence indicating that Tregs mediate tolerance in immune-induced liver injury [17,19], the potential for inducing Tregs by agonist ligands or immunization to reduce immune-mediated liver damage has not been determined. In this study, we examined the protective effect of high-dose gp96 immunization in Con A- and anti-CD137 antibody-induced mouse liver injury models. We found that treatment with gp96 suppressed the symptoms of Con A- and anti-CD137-induced hepatitis. The protection was related to the increased number and enhanced suppressive function of Tregs, resulting in reduced IFN-γ ^+^ CD4^+^ and IFN-γ ^+^ CD8^+^ T cells in the liver and spleen. Moreover, our findings revealed the mechanisms of gp96-mediated enhancement of Treg proliferation and function by activation of a TLR2/TLR4-dependent NF-κB pathway.

Despite a number of reports indicating that the frequencies of Tregs are closely associated with disease progression in HBV-infected patients [4,15,16,35], the function of Tregs in HBV-associated pathogenesis is just beginning to be explored. Tregs and the production of the suppressive cytokine IL-10 are crucial to tolerance induction in a mouse model of Con A hepatitis [19]. Moreover, transient depletion of Tregs in transgenic DEREG mice leads to increased LILs and enhanced early immune-mediated liver damage under AdHBV (adenoviral vector transferring HBV-genome) infection [17]. IL-33 attenuates Con A-induced liver injury by increasing the numbers of Tregs and IL-4-producing CD4^+^ T cells [20]. All of these studies suggest the therapeutic potential of Tregs against immune-mediated liver damage.

In this study, we investigated the protective effect of gp96 immunization in two experimental murine models: Con A-induced hepatitis, which has been extensively used to study T-cell-mediated hepatic injury [21,36]; and anti-CD137-induced hepatitis, which mimics T-cell-induced liver pathogenesis in CHB. We found that a high dose of gp96 enhances Treg proliferation and function, thus restraining T cell activation and pro-inflammatory cytokine expression and thereby enabling gp96 to exert a protective effect. Currently, we only show the prophylactic effect of high dose of gp96 immunization against T-cell mediated liver injury in the murine models of hepatitis, due to the limits of Con A-induced hepatitis which could only last 48 h (Figure 1B). It will be worthwhile to examine the therapeutic efficiency of gp96 immunization against immune-mediated hepatic injury in a context where there already exist significant numbers of activated T cells. Given that the symptoms fulminant hepatitis or acute of chronic liver failure in CHB may last for weeks to months, and gp96-induced protection appears as early as 10 d after gp96 administration, we believe that induction of Tregs by gp96 administration may probably provide an effective strategy for treatment of immune-induced liver injury in fulminant hepatitis.

Murine Tregs express almost all of the identified TLR genes [37,38], and TLR (e.g., TLR2, 4, 7, 8, 9) signaling has a direct impact on Treg function [38,39]. However, the exact roles for specific TLR ligands or agonists in enhancing or reversing Treg function are somewhat controversial [32,37–42], which may be due to the different immune contexts studied. Here, we show that gp96 directly bound to Tregs via interaction with TLR2 and TLR4, thus activating the NF-κB pathway, which promotes Foxp3, IL-10, and TGF-β1 expression. Foxp3 and the suppressive cytokines IL-10 and TGF-β, which play key roles in Treg function, are regulated by NF-κB [43–45]. However, at present, we cannot exclude the possibility that Treg activation following gp96 administration partially results from the interaction with other immune cell types. As systemic administration of high doses of CpGs that bind to TLR9 ligands has been shown to stimulate Tregs by inducing plasmacytoid dendritic cells (pDCs) to express the immunosuppressive enzyme indoleamine 2,3 dioxygenase (IDO) [46], we determined IDO expression in pDCs by real time PCR 24 h after gp96 immunization. The results showed that there was no significant change of IDO expression after gp96 immunization (data not shown), indicating that gp96 activates Tregs not mainly through IDO induction.

The activation of Tregs requires high-dose gp96 stimulation. This may be due to the limited access of gp96 to Tregs, which only account for ~3% of total splenocytes. Another explanation is that the anergy state of Tregs in a normal host requires a high amount of gp96 binding to its surface to activate downstream signaling. Intriguingly, Chandawarkar et al [47] demonstrated that high dose gp96 immunization-induced suppressive CD4^+^CD25^+^ and CD4^+^CD25^-^ T cell populations had similar protective activity against diabetes in the adoptive transfer experiments, which is different from our observation that only CD4^+^CD25^+^ but not CD4^+^CD25^-^ T cell populations exhibited protective effect against Con A-induced liver injury (Figure 2H). The discrepancy may be explained by different duration of adoptive transfer of the T cell subpopulations. It is possible that although the CD4^+^CD25^-^ T cells from high dose gp96-immunised mice did not exhibit immediate suppressive effect in Con A mouse model (as CD4^+^CD25^-^ cells were transferred only one day before Con A treatment), these T cell subpopulations may convert to CD4^+^CD25^+^ T cells *in vivo* upon homeostatic proliferation during the long-term transfer experiments in NOD mouse model (as CD4^+^CD25^-^ cells were transferred into recipients of NOD mice and serum glucose levels were monitored throughout 16 weeks). This deserves further investigation.

Finally, as HBV or liver antigen-specific Tregs may play a key role in suppression of acute exacerbation and hepatic flares in CHB [15,16,48], induction of putative antigen-specific Tregs could effectively suppress immune hyperactivation in fulminant hepatitis. Conceivably, immunization with high dose gp96 complexed with specific antigens may provide an efficient way for induction and activation of antigen-specific Tregs as our previous study indicates that HBV-specific Tregs were possibly generated through gp96 immunization [25]. The possibility is currently under investigation.

In conclusion, we demonstrated that high-dose gp96 immunization elevates the frequency and suppressive function of Tregs, thus protecting mice from liver damage induced by immune hyperactivation. We elucidated, for the first time, how high-dose gp96 activates Tregs. Our work supports the notion that a rational gp96-based therapy could be development against immune-mediated liver destruction in T-cell-mediated immune hyperactivation syndromes.
